# Reliability and Validity of the Swedish Version of the Hull Airway Reflux Questionnaire (HARQ-S)

**DOI:** 10.1007/s00408-016-9937-5

**Published:** 2016-09-16

**Authors:** Ewa-Lena Johansson, Ewa Ternesten-Hasséus

**Affiliations:** 1 Department of Physiotherapy and Occupational Therapy, Sahlgrenska University Hospital/Sahlgrenska, Gothenburg, Sweden; 2 Department of Respiratory Medicine and Allergology, Institution of Medicine, The Sahlgrenska Academy, University of Gothenburg, 413 45 Gothenburg, Sweden

**Keywords:** Chronic cough, Cough hypersensitivity, Questionnaire

## Abstract

**Introduction:**

Cough is a common symptom for which patients seek medical care and is defined as chronic if it has lasted for more than 8 weeks. The Hull Airway Reflux Questionnaire (HARQ) was developed with the aim of eliciting the major component of chronic cough. It comprises 14 items with a maximum total score of 70. A Swedish version (HARQ-S) has previously been developed but not yet formally validated. The aim of the present study was to validate the HARQ-S in terms of instrumental reliability and concurrent validity.

**Methods:**

A total of 67 consecutively selected non-smoking patients with chronic cough and 91 non-smoking allegedly healthy controls were asked to answer two questionnaires, the HARQ-S and a local questionnaire, at two occasions about 3 weeks apart.

**Results:**

The HARQ-S showed good psychometric properties. The patients had significantly higher total scores (*p* < 0.001) compared to the controls, and the questionnaire showed outstanding discrimination ability to distinguish between patients and controls, with an area under the receiver operating characteristic curve of 0.99. Fifty patients and 77 controls completed the HARQ-S twice, showing good test–retest agreement in all items as well as in the total scores in both groups, and without any significant differences over time.

**Conclusion:**

The Swedish version of the HARQ is a valid and reliable questionnaire with good agreement between the two measurements in both patients and controls. The HARQ-S has good reliability and validity and can be used as a diagnostic tool in Swedish-speaking patients with chronic cough.

## Introduction

Cough is a common symptom in patients with different pulmonary diseases and is defined as chronic when it has lasted for more than 8 weeks [1]. Common causes of chronic cough are asthma, chronic obstructive pulmonary disease, gastro-oesophageal reflux disease, and cancer. In many patients, the cause of the chronic cough remains unclear and is refractory to treatment [2, 3]. In a specialist clinic as many as 42 % of patients with cough could be labelled as suffering from chronic cough without any medical explanation, also known as chronic idiopathic cough [2].

The Hull Airway Reflux Questionnaire (HARQ) was developed in the UK, from the Reflux Symptom Index [4], with the aim of eliciting the major components of chronic cough. The English version of the HARQ is validated and has good psychometric properties, internal consistency, and test–retest repeatability. It has a high sensitivity and specificity with a very striking receiver operating characteristic (ROC) curve, and is responsive to treatment [5].

In a pilot study, the HARQ was translated from English to Swedish [6]. In concordance with the English version, the Swedish version (HARQ-S) showed that patients with chronic cough had significantly higher total scores compared to healthy controls, and the ROC curve showed outstanding discrimination ability to distinguish patients with chronic cough from healthy controls [6]. It has also been shown to be responsive to treatment after oral intake of natural capsaicin in patients with chronic cough [7]. The preliminary Swedish version has not yet been formally validated, and there is a need to further assess instrumental reliability of the questionnaire.

The aim of the present study was to validate the HARQ-S in terms of instrumental reliability (degree of agreement, test–retest reliability, and internal consistency reliability) and concurrent validity.

## Methodology

### Study Design

The HARQ-S questionnaire and a local questionnaire were answered at two occasions about 3 weeks apart [6, 8]. At the first opportunity, both questionnaires were handed out in person, and at the second occasion, the questionnaires were sent by postal mail, with a prepaid return envelope. The participants were asked to answer the questions based on their condition as experienced during the previous month. The participants were reminded once within 2 weeks, by telephone, for complementary answers.

Written informed consent was obtained from all participants after they had received information about the study, both verbally and in writing. The study was approved by the Regional Ethical Review Board of Gothenburg, Sweden (number: 542-14).

### Patients

The patients were consecutively selected from May 2011 to June 2012 and from February 2014 to May 2015, when they attended the Department of Asthma and Allergology at the Sahlgrenska University Hospital in Gothenburg, Sweden. All patients had been referred to the clinic due to having had at least 8 weeks of coughing, defined as chronic cough.

### Control Group

The healthy controls, selected to be similar to the patients in age and sex distribution, were recruited over a period of about 9 months. They were subjectively healthy and were recruited among friends and staff at the Sahlgrenska University Hospital. They were screened using questions on cough and airway symptoms. Anyone identified as having problems with cough or a chronic respiratory disease was excluded.

### Questionnaires

#### The Local Questionnaire

The local questionnaire contained questions regarding demographic data (age, gender, and smoking habits), airway symptoms [8], and any change in health status during the past month.

#### The HARQ-S

The HARQ-S is a self-administered questionnaire and consists of 14 items (Table 1). The participants were asked to evaluate how different problems had affected them during the previous month, on a scale of 0–5 (0 = no problem; 5 = severe/frequent problems). The total score of the questionnaire varies from 0 to 70 points. A total score of <13 points is regarded as normal [5].

**Table 1:** Items of the Hull Airway Reflux Questionnaire

Within the last month, how did the following problems affect you? (0 = no problem and 5 = severe/frequent problem)						
Hoarseness or a problem with your voice	0	1	2	3	4	5
Clearing your throat	0	1	2	3	4	5
Excess mucus in the throat, or drip down the back of your nose	0	1	2	3	4	5
Retching or vomiting when you cough	0	1	2	3	4	5
Cough on first lying down or bending over	0	1	2	3	4	5
Chest tightness or wheeze when coughing	0	1	2	3	4	5
Heartburn, indigestion, stomach acid coming up (or do you take medications for this, if yes score 5)	0	1	2	3	4	5
A tickle in your throat, or a lump in your throat	0	1	2	3	4	5
Cough with eating (during or soon after meals)	0	1	2	3	4	5
Cough with certain foods	0	1	2	3	4	5
Cough when you get out of bed in the morning	0	1	2	3	4	5
Cough brought on by singing or speaking (for example, on the telephone)	0	1	2	3	4	5
Coughing during the day rather than night	0	1	2	3	4	5
A strange taste in your mouth	0	1	2	3	4	5
Total score	70	–	–	–	–	–

This is self-administered and has 14 items. Responses to each question can vary from 0 to 5

### Statistical Methods

Descriptive statistics were used to examine the data. Data are presented for continuous variables as mean and standard deviation (SD), mean and 95 % confidence interval (CI), and median and range. Categorical variables are presented in percentage (%) and numbers.

For comparison between groups (patients vs. controls, and males vs. females), Mann–Whitney U-test and unpaired *t* test were used for continuous variables. For calculation of male/female ratio, Fisher’s exact test was used.

To check the suitability of the HARQ-S, the percentages of participants obtaining the lowest possible score of 0 (floor effect) and highest possible score of 5 (ceiling effect) for each item were calculated at the first occasion.

#### Instrumental Reliability

For comparison over time, the Wilcoxon signed-rank test was used for continuous variables, and sign test was used for categorical variables.

For each individual item, test–retest reliability between occasion 1 and occasion 2 is presented as a percentage (%) of decrease/equal/increase degree of agreement [9]. Test–retest reliability for individual question items was also measured using the weighted kappa statistics [10]. The repeatability of the total score was estimated using the method described by Bland and Altman, including calculation of limits of agreement equal to the mean difference of the test–retest values ± twice the SD [11]. Moreover, test–retest reliability for the total score was measured using intraclass correlation coefficient (ICC) [12], and the SD of each participant’s response in total score was calculated using intraindividual standard deviation (IISD) [13].

Internal consistency reliability was expressed as Cronbach’s coefficient alpha (α). A coefficient of >0.70 is considered to be acceptable and satisfactory reliability [14].

#### Concurrent Validity

The ability of the questionnaire to distinguish patients from control subjects was evaluated by constructing a ROC curve [15]. An area under the curve of more than 0.90 indicates that a method has outstanding discrimination ability to distinguish two groups from each other [16].

All tests were two-tailed, and the results were considered significant if *p* < 0.05.

The statistical analyses were carried out using SAS Version 9.3 (SAS Institute, Inc., Cary, NC, USA) and IBM SPSS Version 22 (IMB SPSS. Inc., New York, USA).

## Results

### Participants

The patient group consisted of 67 non-smoking patients (7 men) with a mean age of 50.1 years (SD = 13.8), and the control group of 91 non-smoking, subjectively healthy individuals (18 men), with a mean age of 47.6 years (SD = 13.1). There were no significant differences between the two groups regarding age (data not shown), and no significant difference was found in male/female ratios between the two groups (data not shown).

### The HARQ-S

The median total score of the HARQ-S from the first question occasion was 31.0 (range: 0–65) in the patient group, and 1.0 (range: 0–18) among the controls (*p* < 0.001). The cut-off limit of 13 points was exceeded in 63 patients (94 %; 57 women), and in one female control subject. Among the patients, the median total score for women was 31.0 (range: 2–65) and 31.0 (range: 4–37) for men (NS). The female patients had significantly higher scores for “cough brought out by singing or speaking” (*p* < 0.05) compared to men; otherwise, there were no gender differences. For the female control subjects, the median total score was 1.0 (range: 0–18), and 0 (range: 0–6) (NS) for the men. The female controls had significantly higher scores for “clearing your throat” compared to men (*p* < 0.05), but no other gender differences were found in the control group.

#### Psychometric Properties

The distribution of the HARQ-S was considered to be normal in the patient group. The prevalence of patients with the lowest possible score of 0 (floor effect) was between 4.5 and 57 %, with the lowest prevalence for “clearing your throat” and the highest for “a strange taste in your mouth”. The prevalence of patients with the highest possible score of 5 (ceiling effect) was between 7.5 and 30 %, with the lowest prevalence for “chest tightness and wheeze” and the highest for “coughing during the day rather than night”. The distribution of the control population was highly skewed; 42 out of 91 (46 %) had a total score of 0. The prevalence of controls with the lowest possible score of 0 (floor effect) was between 68 and 99 %, with the lowest prevalence for “clearing your throat” and the highest for “cough with certain food”. The prevalence of controls with the highest possible score of 5 (ceiling effect) was between 1 and 6.6 %, with the lowest prevalence for “coughing during the day rather than night” and the highest for “heartburn”.

#### Instrumental Reliability

Reproducibility was calculated on the subjects who answered the HARQ-S twice. In total, 50 patients (5 men) and 77 controls (15 men) answered the questionnaire at two opportunities. Seventeen patients (16 did not answer, and one had caught a cold) and 14 control subjects (8 did not answer, and 6 had caught a cold) did not participate the second time. The mean duration between the two answering opportunities was 27.2 (SD = 19.2) days in the patient group and 22.9 (SD = 15.2) days among the controls (NS).

Results for individual question items from the first and second occasions, and percentage of agreement between the two occasions are shown in Table 2. In the patient group, equal percentages of agreement between the two occasions ranged from 32 to 70 %, and in the control group from 75.3 to 100 %. There were no significant differences in either group between the individual items or in the total scores between the first and second occasions. Table 3 shows the weighted kappa statistics for each of the 14 items. The lowest weighted kappa value in the patient group was 0.38 (coughing during the day), and the highest was 0.73 (heartburn). The lowest value among the healthy controls was −0.01 (coughing from speaking) and the highest was 0.91 (heartburn).

**Table 2:** Results of the HARQ-S at the first and second occasions, and percentage of agreement between these two occasions, in 50 patients with chronic cough and 77 controls

Patients			Change from occasion 1 to occasion 2		Controls		Change from occasion 1 to occasion 2	
Item	Occasion 1 (*n* = 50)	Occasion 2 (*n* = 50)		*p* value	Occasion 1 (*n* = 77)	Occasion 2 (*n* = 77)		*p* value
Hoarseness								
0	14 (28 %)	13 (26 %)			67 (87 %)	69 (89.6 %)		
1	9 (18 %)	7 (14 %)			5 (6.5 %)	6 (7.8 %)		
2	1 (2 %)	7 (14 %)			4 (5.2 %)	1 (1.3 %)		
3	10 (20 %)	8 (16 %)	Dec 14 (28 %)		1 (1.3 %)	1 (1.3 %)	Dec 4 (5.2 %)	
4	5 (10 %)	8 (16 %)	Equ 25 (50 %)				Equ 72 (93.5 %)	
5	11 (22 %)	7 (14 %)	Inc 11 (22 %)	0.69			Inc 1 (1.3 %)	0.38
Throat_cl								
0	2 (4.1 %)	6 (12.2 %)			53 (68.8 %)	52 (67.5 %)		
1	4 (8.2 %)	6 (12.2 %)			17 (22.1 %)	22 (28.6 %)		
2	8 (16.3 %)	6 (12.2 %)			7 (9.1 %)	3 (3.9 %)		
3	12 (24.5 %)	5 (10.2 %)	Dec 16 (32.7 %)				Dec 10 (13 %)	
4	10 (20.4 %)	12 (24.5 %)	Equ 20 (40.8 %)				Equ 58 (75.3 %)	
5	13 (26.5 %)	14 (28.6 %)	Inc 13 (26.5 %)	0.71			Inc 9 (11.7 %)	1.00
Mucus								
0	5 (10 %)	8 (16 %)			67 (87 %)	65 (84.4 %)		
1	7 (14 %)	2 (4 %)			7 (9.1 %)	10 (13 %)		
2	10 (20 %)	9 (18 %)			3 (3.9 %)	2 (2.6 %)		
3	9 (18 %)	13 (26 %)	Dec 16 (32 %)				Dec 4 (5.2 %)	
4	11 (22 %)	9 (18 %)	Equ 16 (32 %)				Equ 67 (87 %)	
5	8 (16 %)	9 (18 %)	Inc 18 (36 %)	0.86			Inc 6 (7.8 %)	0.75
Retching								
0	18 (36 %)	19 (38 %)			75 (97.4 %)	76 (98.7 %)		
1	12 (24 %)	15 (30 %)			1 (1.3 %)	1 (1.3 %)		
2	4 (8 %)	4 (8 %)			1 (1.3 %)	0 (0 %)		
3	7 (14 %)	5 (10 %)	Dec 17 (34 %)				Dec 2 (2.6 %)	
4	4 (8 %)	4 (8 %)	Equ 22 (44 %)				Equ 75 (97.4 %)	
5	5 (10 %)	3 (6 %)	Inc 11 (22 %)	0.34			Inc 0 (0 %)	0.50
Lying down								
0	15 (30 %)	16 (32 %)			75 (97.4 %)	74 (96.1 %)		
1	8 (16 %)	6 (12 %)			2 (2.6 %)	2 (2.6 %)		
2	12 (24 %)	10 (20 %)			0 (0 %)	1 (1.3 %)		
3	4 (8 %)	8 (16 %)	Dec 12 (24 %)				Dec 2 (2.6 %)	
4	1 (2 %)	3 (6 %)	Equ 25 (50 %)				Equ 72 (93.5 %)	
5	10 (20 %)	7 (14 %)	Inc 13 (26 %)	1.00			Inc 3 (3.9 %)	1.00
Wheeze								
0	14 (28 %)	17 (34 %)			74 (96.1 %)	77 (100 %)		
1	9 (18 %)	10 (20 %)			3 (3.9 %)	0 (0 %)		
2	8 (16 %)	5 (10 %)						
3	7 (14 %)	8 (16 %)	Dec 15 (30 %)				Dec 3 (3.9 %)	
4	9 (18 %)	4 (8 %)	Equ 22 (44 %)				Equ 74 (96.1 %)	
5	3 (6 %)	6 (12 %)	Inc 13 (26 %)	0.85			Inc 0 (0 %)	0.25
Heartburn								
0	26 (52 %)	21 (42 %)			63 (81.8 %)	61 (79.2 %)		
1	4 (8 %)	4 (8 %)			5 (6.5 %)	7 (9.1 %)		
2	2 (4 %)	6 (12 %)			2 (2.6 %)	3 (3.9 %)		
3	4 (8 %)	2 (4 %)	Dec 4 (8 %)		1 (1.3 %)	0 (0 %)	Dec 3 (3.9 %)	
4	2 (4 %)	2 (4 %)	Equ 35 (70 %)		0 (0 %)	0 (0 %)	Equ 70 (90.9 %)	
5	12 (24 %)	15 (30 %)	Inc 11 (22 %)	0.12	6 (7.8 %)	6 (7.8 %)	Inc 4 (5.2 %)	1.00
Tickle								
0	5 (10 %)	12 (24 %)			68 (88.3 %)	74 (96.1 %)		
1	11 (22 %)	5 (10 %)			7 (9.1 %)	1 (1.3 %)		
2	6 (12 %)	7 (14 %)			1 (1.3 %)	1 (1.3 %)		
3	6 (12 %)	6 (12 %)	Dec 16 (32 %)		1 (1.3 %)	1 (1.3 %)	Dec 6 (7.8 %)	
4	9 (18 %)	9 (18 %)	Equ 23 (46 %)				Equ 69 (89.6 %)	
5	13 (26 %)	11 (22 %)	Inc 11 (22 %)	0.44			Inc 2 (2.6 %)	0.29
Eating								
0	19 (38 %)	17 (34 %)			77 (100 %)	77 (100 %)		
1	6 (12 %)	4 (8 %)						
2	5 (10 %)	4 (8 %)						
3	5 (10 %)	7 (14 %)	Dec 10 (20 %)				Dec 0 (0 %)	
4	8 (16 %)	9 (18 %)	Equ 24 (48 %)				Equ 77 (100 %)	
5	7 (14 %)	9 (18 %)	Inc 16 (32 %)	0.33			Inc 0 (0 %)	
Certain_foods								
0	17 (34 %)	17 (34 %)			75 (97.4 %)	75 (97.4 %)		
1	7 (14 %)	6 (12 %)			1 (1.3 %)	1 (1.3 %)		
2	4 (8 %)	4 (8 %)			1 (1.3 %)	1 (1.3 %)		
3	6 (12 %)	8 (16 %)	Dec 11 (22 %)				Dec 2 (2.6 %)	
4	8 (16 %)	9 (18 %)	Equ 27 (54 %)				Equ 74 (96.1 %)	
5	8 (16 %)	6 (12 %)	Inc 12 (24 %)	1.00			Inc 1 (1.3 %)	1.00
Out_of_bed								
0	15 (30 %)	12 (24 %)			73 (94.8 %)	72 (93.5 %)		
1	7 (14 %)	7 (14 %)			2 (2.6 %)	3 (3.9 %)		
2	5 (10 %)	9 (18 %)			2 (2.6 %)	1 (1.3 %)		
3	9 (18 %)	9 (18 %)	Dec 9 (18 %)		0 (0.0 %)	1 (1.3 %)	Dec 2 (2.6 %)	
4	9 (18 %)	8 (16 %)	Equ 26 (52 %)				Equ 73 (94.8 %)	
5	5 (10 %)	5 (10 %)	Inc 15 (30 %)	0.31			Inc 2 (2.6 %)	1.00
Speaking								
0	10 (20.0 %)	9 (18.0 %)			76 (98.7 %)	75 (97.4 %)		
1	2 (4.0 %)	4 (8.0 %)			1 (1.3 %)	0 (0.0 %)		
2	11 (22.0 %)	8 (16.0 %)			0 (0.0 %)	0 (0.0 %)		
3	8 (16.0 %)	9 (18.0 %)	Dec 11 (22.0 %)		0 (0.0 %)	2 (2.6 %)	Dec 1 (1.3 %)	
4	10 (20.0 %)	11 (22.0 %)	Equ 26 (52.0 %)				Equ 74 (96.1 %)	
5	9 (18.0 %)	9 (18.0 %)	Inc 13 (26.0 %)	0.84			Inc 2 (2.6 %)	1.00
Day								
0	5 (10 %)	6 (12 %)			75 (97.4 %)	73 (94.8 %)		
1	3 (6 %)	5 (10 %)			1 (1.3 %)	1 (1.3 %)		
2	6 (12 %)	3 (6 %)			1 (1.3 %)	1 (1.3 %)		
3	10 (20 %)	7 (14 %)	Dec 15 (30 %)		0 (0 %)	1 (1.3 %)	Dec 1 (1.3 %)	
4	12 (24 %)	11 (22 %)	Equ 19 (38 %)		0 (0 %)	0 (0 %)	Equ 72 (93.5 %)	
5	14 (28 %)	18 (36 %)	Inc 16 (32 %)	1.00	0 (0 %)	1 (1.3 %)	Inc 4 (5.2 %)	0.38
Taste								
0	29 (58 %)	27 (54 %)			73 (94.8 %)	75 (97.4 %)		
1	6 (12 %)	7 (14 %)			2 (2.6 %)	2 (2.6 %)		
2	8 (16 %)	3 (6 %)			1 (1.3 %)	0 (0 %)		
3	2 (4 %)	8 (16 %)	Dec 8 (16 %)		0 (0 %)	0 (0 %)	Dec 4 (5.2 %)	
4	1 (2 %)	2 (4 %)	Equ 30 (60 %)		1 (1.3 %)	0 (0 %)	Equ 72 (93.5 %)	
5	4 (8 %)	3 (6 %)	Inc 12 (24 %)	0.50	0 (0 %)	0 (0 %)	Inc 1 (1.3 %)	0.38
Total score	31.6 (13.0)	31.7 (14.3)	0.08 (8.1)		1.9 (2.6)	1.8 (3.5)	−0.08 (2.4)	
	30.0 (4.0–64.0)	33.0 (4.0–59.0)	−0.5 (−20.0–19.8)		1.00 (0.0–11.0)	0.0 (0.0–20.0)	−0.0 (−5.0–14.0)	
	*n* = 50	*n* = 50	*n* = 50	0.96	*n* = 77	*n* = 77	*n* = 77	0.17

For categorical variables, *n* (%) is presented. For continuous variables mean (SD)/median (range)/*n* = is presented. For comparison over time, the Wilcoxon signed-rank test was used for continuous variables and sign test was used for categorical variables

*Dec* decrease, *Equ* equal, *Inc* increase, *n* number, *SD* standard deviation

**Table 3:** Weighted kappa statistics (95 % CI) for each item, in patients with chronic cough and controls

Item	wkappa (patients *n* = 50)	wkappa (controls *n* = 77)
Hoarseness	0.60 (0.46–0.74)	0.72 (0.47–0.96)
Throat_clearing	0.43 (0.25–0.62)	0.51 (0.34–0.68)
Mucus	0.45 (0.30–0.59)	0.54 (0.25–0.82)
Retching	0.53 (0.37–0.70)	0.49 (0.15–0.84)
Lying_down	0.57 (0.41–0.73)	−0.03 (−0.05–0.00)
Wheeze	0.51 (0.34–0.67)	−0.00 (−0.00–−0.00)
Heartburn	0.73 (0.60–0.86)	0.91 (0.83–0.98)
Tickle	0.54 (0.38–0.70)	0.30 (0.06–0.54)
Eating	0.60 (0.45–0.74)	(–)
Certain_foods	0.59 (0.43–0.76)	0.32 (−0.09–0.73)
Out_of_bed	0.65 (0.53–0.78)	0.55 (0.16–0.94)
Speaking	0.64 (0.51–0.78)	−0.01 (−0.02–0.01)
Day	0.38 (0.20–0.56)	0.27 (−0.15–0.69)
Taste	0.64 (0.49–0.80)	0.18 (−0.14–0.50)

*CI* confidence interval, *n* number, *(–)* in both occasions no occasions of “eating” was found, and weighted kappa could not be calculated

Limits of agreement, IISD, and ICC of the total score are presented in Table 4. The standard deviation of the differences was 8.12 in the patient group and 2.45 in the control group, the ICC showed high agreement in both groups (0.83 and 0.68, respectively), and the IISD was 5.69 in the patient group and 1.72 in the control group.

**Table 4:** Limits of agreement, intraclass correlation coefficient (ICC), and intraindividual SD (IISD) in patients with chronic cough and controls

Variable	Difference Occasion 2−Occasion 1		Intraclass correlation coefficient (ICC) (95 % CI)	Intraindividual SD (IISD)
	Mean (95 % CI limits of agreement) (SD) median (range)	Systematic changes *p* value		
Total score (patients) (*n* = 50)	0.076 (−15.847; 15.999) (8.124) −0.500 (−20.000–19.800)	0.9584	0.83 (0.71; 0.90)	5.69
Total score (controls) (*n* = 77)	−0.078 (−4.887; 4.731) (2.454) 0.000 (−5.000–14.000)	0.1736	0.68 (0.54; 0.79)	1.72

Wilcoxon signed-rank test is used to test the difference. For difference mean (95 % CI, limits of agreement)/(SD)/median (range)/*n* = is presented

*CI* Confidence interval, *ICC* intraclass correlation, *IISD* intraindividual standard deviation, *n* number, *SD* standard deviation

The Cronbach’s alpha coefficient, representing the internal consistency reliability, was 0.82 in the patient group and 0.64 in the control group.

#### Concurrent Validity

**Fig. 1:**
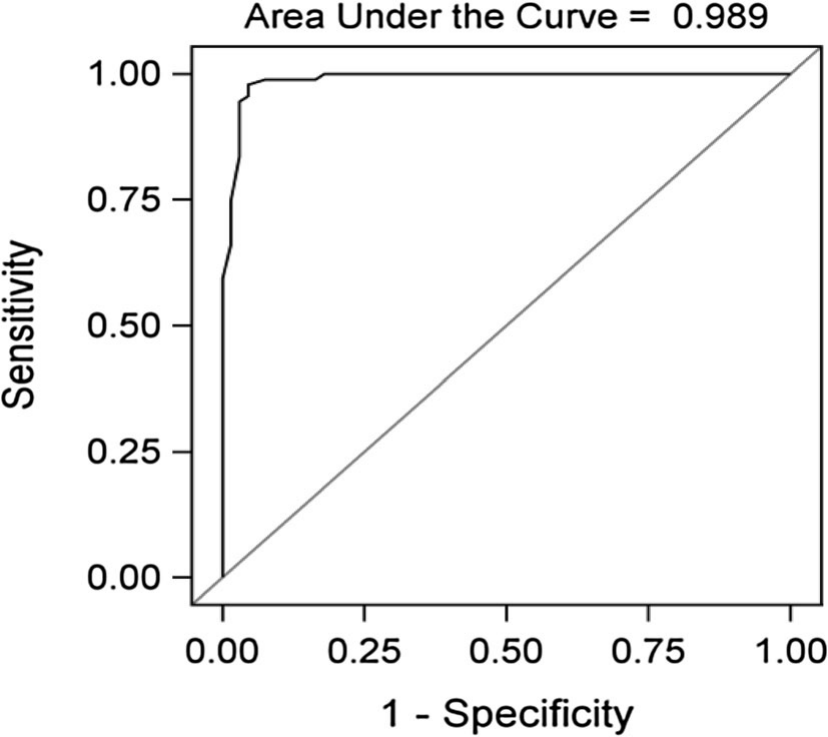
ROC curve for the ability to distinguish patients with chronic cough from control subjects

The area under the ROC curve was 0.99, which corresponds to outstanding discrimination ability between patients and controls (Fig. 1).

## Discussion

In most ways, the findings of the HARQ-S are consistent with those from the original English version. The main results of the Swedish version can be summarized as follows. First, the results showed that the HARQ-S has good psychometric properties. Second, the patients with chronic cough had significantly higher total scores compared to the control group, and the area under the ROC curve showed outstanding discrimination ability to distinguish between patients with chronic cough and controls. Third, for the total score, the test–retest agreement was considered to be good in all items, without any significant differences over time, and the test–retest reliability was considered to be good. Further, the HARQ-S had good internal consistency reliability.

The HARQ-S showed good psychometric properties, and no single response was given by more than 57 % of the chronic cough patients, in line with the results from the original HARQ study [5]. Further, the HARQ-S results in the present study demonstrated no differences in the total scores between women and men with chronic cough in disparity to the original study [5] and to the results from a former Swedish pilot study [6], both showing significantly higher total scores for women than men. One possible reason for not finding any gender differences in the present study may be the sparse number of male patients included in comparison to the previous studies [5, 6]. Likewise, we found no gender differences among the healthy controls.

Test–retest reliability can be evaluated using percentage of agreement [9] and weighted kappa statistics [10]. In the original version of the HARQ, test–retest reliability was studied using weighted kappa statistics in a group of patients with chronic cough but not among controls [5]. In this study, we evaluated test–retest reliability in both groups, and we used both the percentage agreement statistics and the weighted kappa statistics. The results showed that the percentages of agreement were satisfactory in both groups. According to Viera et al., a weighted kappa value of >0.40 is suggested to show moderate agreement [10], and in the English-speaking patient group, the weighted kappa values ranged from 0.40 to 0.79 [5], whereas the present results ranged from 0.38 to 0.73, demonstrating almost the same kappa values. In accordance with Morice et al., we found that among the patients, the item “cough during the day rather than the night” had the lowest kappa value (0.40 and 0.38, respectively) [5]; otherwise, all kappa values were above 0.40. Among the controls, the present weighted kappa results were in some items close to or below zero (cough related to lying down, wheeze, eating, and speaking), but on the other hand, these items had a high percentage of equal agreement, with values above 93 %. The findings of low weighted kappa values in combination with a high percentage of agreement, are paradox, but can be explained by the fact that it is impossible to calculate kappa if the percentages of agreement are close to 0 % or close to 100 % [17, 18].

The standard deviation of the differences showed almost the same results in the present study as in the original study (8.12 and 8.23, respectively) [5]. Further, the ICC was used for analysing test–retest reliability. An ICC value >0.4 is generally regarded as a moderate correlation and >0.75 as a strong correlation [12]. The ICC of the HARQ-S was considered to be good, with high values in both patients and controls (0.83 and 0.68, respectively). The IISD, describing the within-person variation, was likewise good in both groups, though somewhat lower among the control subjects.

The internal consistency reliability, expressed as Cronbach’s coefficient α, reflecting the HARQ’s ability to indicate the extent to which items are related, was high in the patient group (0.82) but lower in the control group (0.64). This is in line with the study by Morice et al. showing a Cronbach’s α coefficient of 0.81 in patients with chronic cough [5].

In accordance with other studies by Morice et al. [5] and Ternesten-Hasséus et al. [6], the ROC curve in the present study showed outstanding discrimination ability to distinguish patients with chronic cough from healthy controls.

Cough is a worldwide major medical problem, being the cardinal symptom not only of many severe diseases but also of different, quite harmless conditions. Diagnosing cough requires a battery of examinations, and this questionnaire could be a useful tool in discriminating among different kinds of cough. Morice et al. postulated that a majority of patients with chronic cough represent a discrete clinical entity, the newly established “Cough hypersensitivity syndrome” [19, 20]. Within the syndrome, there are different phenotypes, but it has been suggested that a majority of the patients suffer from a precipitant of non-acid reflux, with gaseous mist which causes inflammation and gives rise to hypersensitivity and coughing [19, 20]. In accordance with this, we suggest entitle the questionnaire “The Hull Cough Hypersensitivity Questionnaire”.

## Conclusions

In conclusion, the present results conform well to the original questionnaire by Morice et al. [5], and we found in the present study a good agreement between the two measurements in both patients and controls. The HARQ-S has good reliability and validity and can be used as a diagnostic tool in Swedish-speaking patients with chronic cough.
